# WIC Participation and Breastfeeding after the 2009 WIC Revision: A Propensity Score Approach

**DOI:** 10.3390/ijerph16152645

**Published:** 2019-07-24

**Authors:** Kelin Li, Ming Wen, Megan Reynolds, Qi Zhang

**Affiliations:** 1 Department of Sociology, California State University-Dominguez Hills, Carson, CA 90747, USA; 2 Department of Sociology, University of Utah, Salt Lake City, UT 84112, USA; 3 School of Community and Environmental Health, Old Dominion University, Norfolk, VA 23529, USA

**Keywords:** women, infants, and children (WIC), breastfeeding, NHANES, propensity score

## Abstract

In this study, we examined the association between participation in the Special Supplemental Nutrition Program for Women, Infants, and Children (WIC) and breastfeeding outcomes before and after the 2009 revisions. Four-thousand-three-hundred-and-eight WIC-eligible children younger than 60 months were included from the 2005–2014 National Health and Nutrition Examination Survey (NHANES). We compared two birth cohorts with regard to their associations between WIC participation and being ever-breastfed and breastfed at 6 months. We estimated the average effect of the treatment for the treated to assess the causal effect of WIC participation on breastfeeding based on propensity score matching. The results showed that WIC-eligible participating children born between 2000 and 2008 were significantly less likely than WIC-eligible nonparticipating children to ever receive breastfeeding (*p* < 0.05) or to be breastfed at 6 months (*p* < 0.05). Among children born between 2009 and 2014, WIC-eligible participating children were no longer less likely to ever receive breastfeeding compared to WIC-eligible nonparticipating children; the gap remained in breastfeeding at 6-months (*p* < 0.05). The disparities in prevalence of ever breastfed between WIC-eligible participants and nonparticipants have been eliminated since the 2009 WIC revision. More efforts are needed to improve breastfeeding persistence among WIC-participating mother–infant dyads.

## 1. Introduction

The Special Supplemental Nutrition Program for Women, Infants, and Children (WIC) is a federally supported nutrition assistance program targeting low-income women, infants, and children in the United States (U.S.). This program was serving more than a quarter of the pregnant women and half of the newborn infants nationwide in 2016, at an annual cost of about $6.2 billion [1]. One of the program priorities is to support breastfeeding among WIC-participating mothers and infants, since breastfeeding is a crucial maternal practice with both short-term and long-term health benefits [2]. Although the WIC program has been applauded for its overarching goal of breastfeeding support, the availability and distribution of formula to nonfully-breastfed infants have continuously raised questions about the association between WIC participation and breastfeeding outcomes. Previous studies have found that breastfeeding rates were lower among eligible WIC participants than among eligible WIC nonparticipants. This finding has emerged from studies including the Pregnancy Nutrition Surveillance System [3], the National Immunization Survey [4,5,6], the Infant Feeding Practices Survey II [7,8], the Early Childhood Longitudinal Study-Birth Cohort [9,10], the Ross Laboratories Mothers’ Survey [11], and the Feeding Infants and Toddlers Study [12], as well as several primary data collection efforts [13,14]. While the preponderance of evidence has suggested a negative relationship between WIC participation and breastfeeding initiation and duration, some studies had contradictory findings, showing WIC participation as a positive factor for breastfeeding. These studies were largely specific to certain states, such as Texas [15], South Carolina [16] and California [17], with notable exceptions at the national level [18].

The veracity of the “WIC effect” research has been challenged, however, by the possibility of unobserved heterogeneity across participant groups. Some WIC participants may be systematically different than eligible nonparticipants in ways that affect the likelihood of breastfeeding [19,20,21]. For example, the financial benefits of free formula offered through WIC may provide a stronger enrollment incentive to women who plan to feed with formula, rather than breast milk. Differences in social [22,23], cultural [24] and political [25] barriers to breastfeeding have also been documented. Therefore, the lower prevalence of breastfeeding among WIC recipients may be an artifact of this selection bias whereby other factors confound the relationship between WIC program participation and breastfeeding outcomes.

Theoretically the gold standard to obviate selection bias is the experimental design, e.g., randomizing eligible individuals into WIC or non-WIC groups. However, ethical and legal factors make such an experimental design difficult to implement. Therefore, alternative methods must be used to address selection bias and, thereby establish causality between program participation and breastfeeding outcomes. Propensity score matching is one of the most commonly used alternative causal identification strategies. This quasi-experimental technique creates comparable groups of participants and nonparticipants to examine the effects of WIC participation on breastfeeding outcomes and better isolate the treatment effect from the effect of potentially overlooked confounders. This approach has been used in a limited number of studies assessing the link between WIC participation and breastfeeding. For example, using propensity score analysis with data from the Child Development Supplement of the Panel Study of Income Dynamics, Jiang et al. found null and, in some instances, weakly positive effects of WIC participation on breastfeeding [26]. Gregory et al. similarly found null WIC effects on the odds of breastfeeding at three months postpartum using samples matched on sociodemographic and breastfeeding-specific factors, such as prenatal breastfeeding intentions, attitudes towards breastfeeding, and perceived social support for breastfeeding [27]. Taken together, these studies provide further suggestion that the inverse relationship between WIC participation and breastfeeding could be driven by differential selection into the program.

To increase breastfeeding rates among WIC participants, the WIC program implemented a comprehensive food package revision in 2009, the first major change in the WIC food package in four decades [28,29]. Following this revision, the program aimed to promote breastfeeding by raising food benefits for exclusively breastfeeding mothers and reducing the amount of formula for partially breastfeeding mothers. For example, fully breastfeeding mothers can receive 1 lb. (0.45 kg) cheese and 30 oz. (0.85 kg) canned fish a month, which is not available for partially breastfeeding mothers or non-breastfeeding mothers. Fully breastfed infants can receive twice infant fruits and vegetables (256 oz., or 7.26 kg, a month) than partially breastfed and fully formula fed infants (128 oz., or 3.63 kg, a month). Moreover, only fully breastfed infants can receive 77.5 oz. (2.2 kg) infant food meat a month. The effectiveness of this initiative is not yet clear [30]. Several regional studies have conducted pre-post comparisons, but results regarding the impact of this revision on breastfeeding outcomes were mixed. While some found an increase following the 2009 WIC revision [31,32] in breastfeeding prevalence or issuance of “fully breastfeeding” food packages [33], others found no change or even more full-formula package issuance [34,35,36].

All these studies were at local, state, or regional levels; whether the 2009 revision has successfully increased breastfeeding rates among WIC-participants at the national level remains largely unknown. In a previous study, we used national data for 1999–2014 and found that the gap in ever-breastfeeding between WIC participants and eligible nonparticipants was reduced after the 2009 revision while the gap in breastfed at 6-months remained [37]. However, since we did not address potential “selection bias” into the WIC program in this previous study, no causal conclusions can be drawn regarding the relationship between WIC participation and breastfeeding outcomes.

In the current study, we applied propensity-score matching to account for selection bias in the WIC participation/breastfeeding relationship. We used nationally representative data from the National Health and Nutrition Examination Survey (NHANES) and compared WIC effects between children of two different birth cohorts: those born between 2000 and 2008 and those born between 2009 and 2014. Using this approach, we were able to assess whether the association between WIC participation and breastfeeding among the WIC-eligible changed after the 2009 revision while attending to the risk of selection bias.

## 2. Materials and Methods

### 2.1. Data

NHANES is a nationally representative, cross-sectional survey that gathers data every two years on the nutrition and health status of U.S. civilian populations of all ages. NHANES adopts a stratified, multistage probability cluster sampling design and follows standardized protocols for interviews and physical examinations. More detailed descriptions of sampling methodology, survey design, and interview procedures can be found on the NHANES website. NHANES is listed by the Centers for Disease Control as one of the key data sources to monitor breastfeeding prevalence and trends in the U.S. It also includes household information about participation in federal food assistance programs such as WIC and the Supplemental Nutrition Assistance Program (SNAP), formerly known as the Food Stamp Program.

Following the age restrictions for WIC participation, we included children younger than 5 years old (or 60 months). Thus, the age range of our analytical sample was from 0 to 59 months old, including 2770 WIC-eligible children born between 2000 and 2008 and 1538 WIC-eligible children born between 2009 and 2014.

### 2.2. Measurement

Two breastfeeding outcomes were analyzed in this study: ever-breastfed and breastfed at 6-months. Ever-breastfed was determined by a question asking respondents, “Was the child ever breastfed or fed breast milk?” Response options included “Yes” and “No”. Among respondents who indicated “Yes” to ever-breastfed, a follow-up question was asked: “How old was the child when the child completely stopped breastfeeding or being fed breast milk?” Based on this information, we further constructed a binary measure of breastfed at 6 months (Yes/No), which was only applicable to children of at least 6 months old.

We used information on WIC eligibility and WIC participation to distinguish the two comparison groups in propensity score analysis: WIC-eligible participants (treatment group) and WIC-eligible nonparticipants (control group). WIC eligibility was primarily determined according to a child’s family income–poverty ratio, which was defined as the ratio of household income to the poverty guidelines given a certain family size and year. Based on WIC program rules, we coded only women, infants, and children with a household income below or equal to 185% of the federal poverty line (i.e., income–poverty ratio ≤ 1.85) as eligible for WIC. WIC participation was based on the question asking how long a child had received benefits from the WIC program (in months). Children whose participation period was greater than zero were defined as WIC participants.

Birth cohorts of 2000–2008 and 2009–2014 were created based on the survey year of NHANES and the children’s age in months. Specifically, the 2000–2008 cohort included children younger than 60 months in 2005–2008 waves of NHANES. The 2009–2014 cohort included children younger than 12 months in the 2009–2010 NHANES, children younger than 36 months in the 2011–2012 NHANES, and children younger than 60 months in the 2013–2014 NHANES, so all infants and children in the 2009–2014 cohorts were born in or after 2009. Children in the 2009–2014 NHANES who were born before 2008 were included in the birth cohort of 2000–2008.

Variables used to estimate propensity scores included several key demographic characteristics of the child: age in months, gender (male and female), and race/ethnicity (non-Hispanic white, non-Hispanic black, Mexican American, other Hispanics, and other race). Additional variables pertained to the child’s family characteristics: marital status (married/living with partner, widowed/divorced/separated, and never married), educational attainment of the household reference person (less than high school, high school graduate, some college, and college graduate or higher), as well as household income to poverty ratio, which was defined as the household income divided by the applicable federal poverty line. We also included information about whether the household had ever received food stamps in the analyses because participation in other social welfare programs has been shown to be linked to WIC participation [37,38].

### 2.3. Analytical Approach

Although conventional regression models have been used to examine the association between WIC participation and breastfeeding, a major methodological challenge is that causal inference is prohibited by observational studies of cross-sectional data. Propensity score analysis provides an alternative approach that can aid in causal inference when an experimental design is not possible [39]. This approach uses variables available in the observational data to estimate a propensity score for each individual subject, defined as the probability of being in the treatment group (being exposed, i.e., participating in WIC) versus the probability of being in the control group (not being exposed, i.e., not participating in WIC) given this vector of observed variables. Then, by matching individuals with the same propensity for the treatment, but different treatment statuses, to one another, a sample is generated wherein we have two groups that can be directly compared. In essence, this technique mimics the experimental design that is regarded as the gold standard for assessing causal inference.

Our analysis followed the procedures recommended by Oakes and Johnson [40]. First, we estimated propensity scores for each child using a logistic regression model that predicted the likelihood of his or her participating in WIC based on a set of individual and household characteristics. After a propensity score was estimated for each child, we examined the overlap in propensity scores between the two treatment groups (i.e., WIC-eligible participants and WIC-eligible nonparticipants). Once the overlap was determined to be sufficient, we then proceeded with nearest neighbor matching using a range of 0.01. Nearest neighbor was our preferred matching choice because of the transparency afforded by matching only within a predetermined range [40]. We also ensured that all observed factors were balanced between the two groups within each propensity score stratum [39]. In the final step, we estimated the average effect of the treatment for the treated (ATT) to assess the causal effect of WIC participation [41]:

*E* [(*Y*_1_−*Y*_0_) | *X*, *W* = 1] (1)

where *Y*_1_ indicated the two breastfeeding outcomes (i.e., ever-breastfed and breastfed at 6 months) that would have resulted if a WIC-eligible child had participated in the WIC program and *Y*_0_ indicated the breastfeeding outcomes if the same child had not participated in WIC. The treatment effect of participation on the two breastfeeding outcomes was expressed as *Y*_1_ − *Y*_0_, conditional on covariates *X*. *W* denoted the causal factor or treatment, which in our study would be WIC participation; *W* = 1 indicated that the treatment effect focused on the treated. We chose to estimate ATT over other treatment effects as ATT is more relevant in policy contexts [42], which is the focus of our study. To compare the relationship between WIC participation and breastfeeding before and after the 2009 WIC revision, we estimated the ATT among two birth cohorts, namely 2000–2008 and 2009–2014. All analyses were conducted in Stata 14. The study was approved by the Institutional Review Board at Old Dominion University.

## 3. Results

Sample characteristics for the two birth cohorts are presented in Table 1 along with *p*-values from significance tests of difference before and after the 2009 WIC revision. Of all WIC-eligible children in the 2000–2008 birth cohort, 82.64% participated in WIC program; the participation rate was higher at 87.65% among the 2009–2014 cohort, and this difference was statistically significant (*p* < 0.001). Comparing the demographic and family characteristics between the two periods, eligible children of the 2000–2008 cohort were older (*p* < 0.001), were more likely to be white or Mexican (*p* < 0.001) and lived in households where the reference person had less education (*p* < 0.001). Their family income was comparable to that of the 2009–2014 cohort, but they were less likely to receive food stamps (*p* < 0.001).

**Figure 1:**
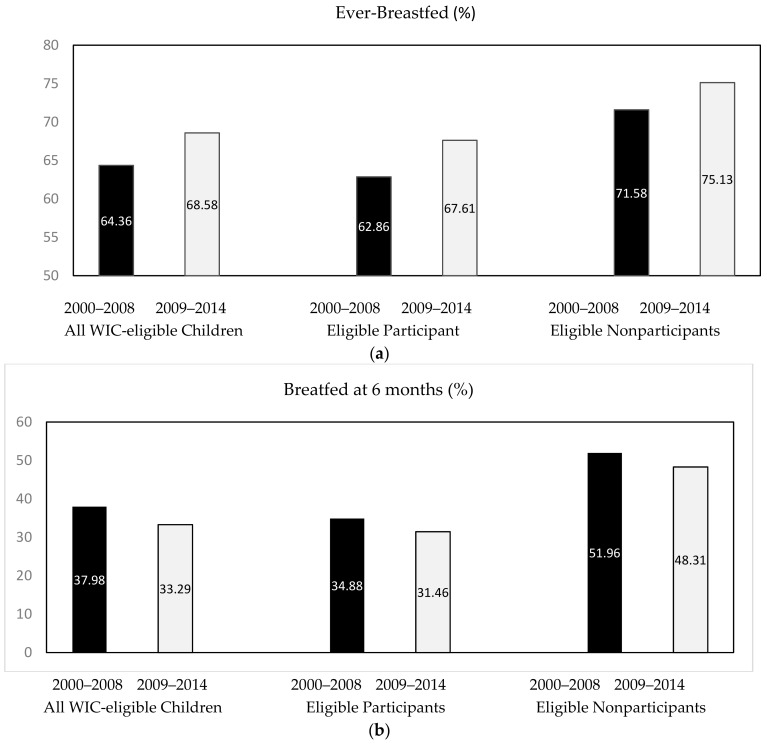
Breastfeeding rates among NHANES children born in 2000–2014. (**a**) Rates of ever-breastfed among WIC-eligible children by birth cohort and participation status. (**b**) Rates of breastfed at 6 months among WIC-eligible children by birth cohort and participation status.

Figure 1A shows that ever-breastfed rates increased between 2000 and 2008 and 2009 and 2014 among both WIC-eligible participating (from 62.86% to 67.61%; *p* = 0.004) and nonparticipating children (from 71.58% to 75.13%; *p* = 0.348), while participating children had significantly lower rates than nonparticipating children (*p* < 0.001). Regarding breastfed at 6 months, Figure 1B shows the opposite trends among WIC-eligible participating and nonparticipating children. Infants breastfed at 6 months had dropped slightly between 2000 and 2008 and 2009 and 2014 among both participating (from 34.88% to 31.46%; *p* = 0.119) and nonparticipating children (from 51.96% to 48.31%; *p* = 0.549). Overall, participating children still had significantly lower breastfeeding rates at 6 months compared to nonparticipants (*p* < 0.001).

**Figure 2:**
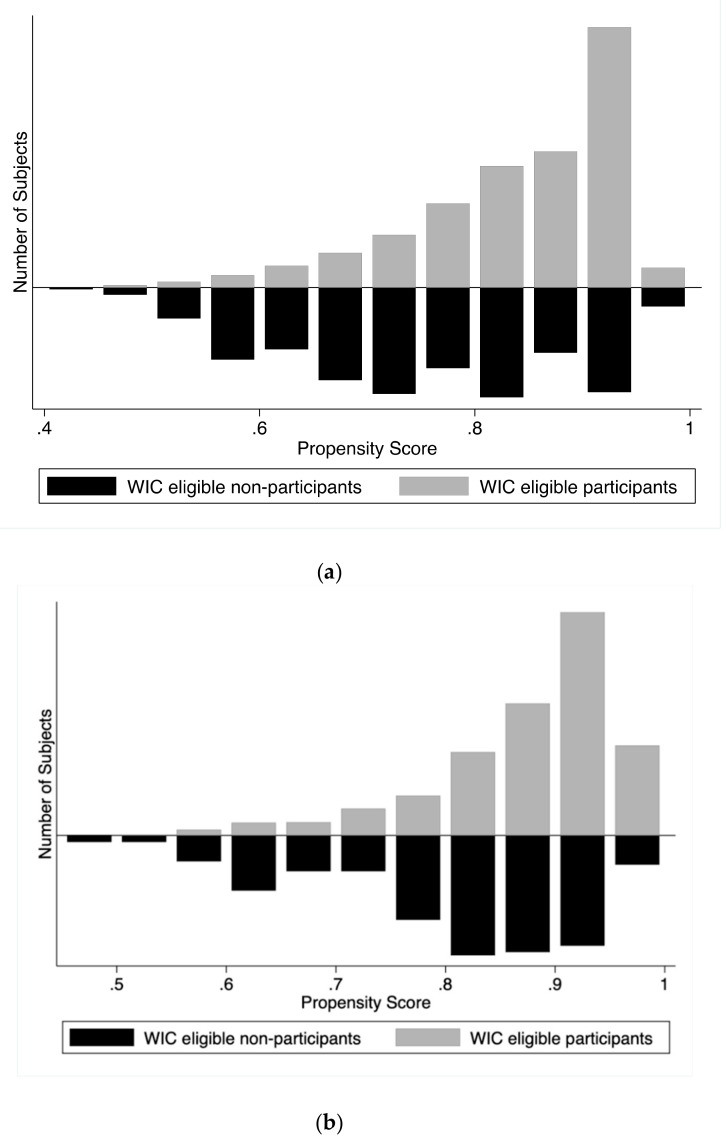
Distributions of NHANES children born in 2000–2014 based on propensity score. (**a**) Overlap in propensity score by WIC participation, birth cohort 2000–2008. (**b**) Overlap in propensity score by WIC participation, birth cohort 2009–2014.

Turning to the propensity score analyses, Figure 2A illustrates the propensity score overlap between the two comparison groups, WIC-eligible participants (treatment group) and WIC-eligible nonparticipants (control group) for birth cohort 2000–2008. Figure 2B illustrates this overlap for birth cohort 2009–2014. As an indication of selection bias, the overlap shows that WIC-eligible participating children had a higher propensity for participating in the WIC program during both periods. A test of the balancing property of propensity scores suggested there was sufficient overlap in the distribution of propensity scores.

Table 2 presents the estimated average effect of the treatment (i.e., WIC participation) for the treated (ATT) after propensity score matching. Consistent with previous evidence using regression methods, our estimates after propensity score matching show that WIC-eligible participating children are significantly less likely than eligible nonparticipating children to ever receive breastfeeding (*p* < 0.05) or to receive breastfeeding at 6 months (*p* < 0.05) among the cohort of 2000–2008. Specifically, in our matched sample, ever-breastfed rates were 66% among participating children and 74% among nonparticipating children; breastfeeding rates at 6 months were 40% among participants and 51% among nonparticipants.

However, in the matched sample for the 2009–2014 cohort, although participating children are still less likely than nonparticipants to ever receive breastfeeding (69% vs. 73%), this difference is no longer statistically significant. Without propensity score matching, this difference would have remained significant; that is, 69% among participants versus 76% among nonparticipants (*p* < 0.05). At the same time, the number of infants breastfed at 6 months is still significantly lower among participants (32%) than among nonparticipants (45%) in the matched sample (*p* < 0.05).

Overall, the above results from the propensity score matching analyses suggest that WIC-participating children were no longer significantly less likely to ever receive breastfeeding than eligible nonparticipating children after the 2009 revision, while breastfeeding at 6 months remained significantly lower among WIC-eligible participants than among nonparticipating children across both periods. The change from the 2000–2008 cohort to the 2009–2014 cohort in terms of having ever been breastfed would not have been detected without propensity score analysis.

## 4. Discussion

This study investigated whether participating in WIC led to lower breastfeeding incidence among U.S. children in two time periods, using propensity score matching to address potential selection bias. By comparing two NHANES birth cohorts, we asked whether the association between WIC participation and breastfeeding changed after the 2009 revision. Our results provide partial support for the notion that the WIC 2009 food package revision reduced breastfeeding disparities among WIC-eligible children. As shown, after the 2009 revision, WIC-participating children were no longer less likely to ever receive breastfeeding compared to eligible nonparticipating children. But their 6-month breastfeeding rate remained significantly lower than for their nonparticipating counterparts, even when controlling for selection bias.

To our knowledge, this is the first study to use a propensity score approach to assessing the WIC participation effect on breastfeeding after the 2009 WIC food package revision. Most studies to date have revealed that breastfeeding rates were generally lower among WIC participants than among WIC nonparticipants [3,4,5,6,7,8,9,10,11,12,13,14]. Yet these studies rely on data and methodological strategies that do not permit comparison of breastfeeding initiation and duration between individuals for whom only the WIC “treatment” varied. The small number of studies that employed matching techniques to equalize the characteristics of WIC participants and (eligible) nonparticipants cast doubt on the conclusions generated under naïve OLS regressions [26,27]. In terms of breastfeeding outcomes, our results were in line with these studies in that WIC participants had lower rates of being ever-breastfed before the 2009 revision, but similar rates after the revision. By using propensity score matching to create a sample of WIC-eligible participating and nonparticipating children that shared similar individual and household socioeconomic characteristics, we sought to minimize the selection effects that are likely to have biased past estimates. Therefore, we are considerably more confident in the results yielded by the observational NHANES data used in the current study.

Our results partially support the positive impact of the 2009 WIC food revision change on breastfeeding by comparing two time periods before and after the revision. A recent review has identified mixed evidence regarding the impact of this revision on breastfeeding outcomes [30]. Other work has reported evidence suggesting more promising trends since the 2009 revision. For example, a study of a predominately Latina sample in Los Angeles County, California, documented improvement in breastfeeding outcomes, as the prevalence of exclusive breastfeeding had nearly doubled [31]. A report sponsored by the Office of Policy Support, Food and Nutrition Service, US Department of Agriculture documents that multiple studies funded by the Food and Nutrition Service have provided direct or indirect evidence supportive of the 2009 federal policy changes’ positive impact [32].

This study complements the existing literature by establishing the causality of WIC participation and breastfeeding outcomes and by comparing this effect before and after the 2009 WIC revision. On the one hand, the WIC effect on being ever-breastfed became insignificant after the 2009 WIC food package revision. One the other hand, being breastfed at 6 months remained significantly lower among WIC participants. As shown, 6-month breastfeeding rates were much lower compared to those of the ever-breastfed; this pattern is understandable due to the challenges associated with breastfeeding per se. It is noteworthy that the 6-month breastfeeding rate dropped from 2005–2010 to 2011–2014 among the WIC participants but not among the nonparticipants in our sample. Although the USDA aimed to encourage breastfeeding and nutrition improvement in the 2009 revision, there is also evidence showing that more WIC mothers of new infants have received the full formula package since the 2009 revision [36]. After that revision, partially breastfeeding mothers could only receive up to one can of formula in the first month, although non-breastfeeding mothers could still have a full formula package. Therefore, it is likely that partially breastfeeding mothers declared themselves non-breastfeeding to have full access to the formula. More research is needed to examine the specific components of the 2009 food package revision and better understand the inter-relationships among the 2009 revision, WIC participation, and breastfeeding outcomes. Moreover, additional factors including fear of public charge and immigration policies may insert influences on their decision to participate among WIC-eligible population. Consideration should also be given to identify barriers and challenges of not only initiating but also continuing breastfeeding practice.

This study is not without limitations. First, our key breastfeeding outcomes are based on self-reported measures; recall bias could have influenced our findings. There is also a possibility to self-report non breastfeeding once participants switch to full formula food package, so that they do not appear to report partially breastfeeding. Second, by creating a matched sample based on propensity scores, we induced a reduction in sample size across both time periods, affording less statistical power with which to detect significant findings. Finally, although propensity score matching overcomes many of the limitations of cross-sectional data, causal inference can be further strengthened with the use of analytic techniques that capitalize on exogenous variation and multiple observations. For example, WIC participation has been instrumented with local food prices [43], WIC program characteristics [18], and WIC clinic availability during pregnancy [44] to generate a measure of WIC participation that is not correlated with the error term. Other studies have applied fixed effects models that treat the individual as their own control using longitudinal data [9,18,45]. Lastly, there is potentially great utility with difference-in-difference models that compare changes over time in treated and untreated groups. In one study examining the policy impact of the Food Stamp program, Hoynes et al. compare metabolic syndrome among adults who did and did not have access to the Food Stamp program during its piecemeal county-level rollout between 1961 and 1975 [46]. More research is needed to confirm the effect of the 2009 WIC package revision on participants’ breastfeeding outcomes.

## 5. Conclusions

This study makes a unique contribution to the literature by analyzing nationally representative cohort data and using propensity score matching to generate novel findings about the association between WIC participation and breastfeeding outcomes. Results from this study should serve as evidence of the success as well as challenges associated with reforms of this major federal nutrition assistance program.

## Figures and Tables

**Table 1:** Descriptive statistics of WIC-eligible children by birth cohort.

Variables	Birth Year 2000–2008	Birth Year 2009–2014	*p-Value*
*Breastfeeding*			
Ever-breastfed	64.36%	68.58%	0.005
Breastfed at 6 months	37.98%	33.29%	0.024
*WIC Participation*			0.000
Eligible participants	82.64%	87.13%	
Eligible nonparticipants	17.36%	12.87%	
*Child Characteristics*			
Age (months)	28.54 (17.44)	19.74 (15.97)	0.000
Male	52.45%	49.54%	0.067
Race			0.000
White	25.31%	22.63%	
Black	22.20%	27.63%	
Mexican	36.43%	26.92%	
Other Hispanic	9.49%	13.00%	
Other races	6.57%	9.82%	
*Family Characteristics*			
Marital status			0.616
Married/living with partner	66.36%	65.19%	
Widowed/divorced/separated	15.25%	15.16%	
Never married	18.40%	19.65%	
Educational attainment			<0.001
Less than high school	43.74%	34.31%	
High school graduate/GED	28.30%	29.00%	
Some college/AA degree	21.98%	29.26%	
College graduate or higher Income–poverty ratio	5.98% 0.87 (0.45)	7.43% 0.85 (0.47)	0.084
Ever received food stamp (Yes)	53.61%	63.20%	<0.001
Sample Size (N)	2770	1538	

Note: Standard deviations are in parentheses.

**Table 2:** Average treatment effect for the treated for breastfeeding outcomes between WIC-eligible participants and nonparticipants by birth cohort.

	Birth Year 2000–2008					Birth Year 2009–2014				
Outcomes	WIC	Non-WIC	Diff	SE	t-statistic	WIC	Non-WIC	Diff	SE	t-Statistic
**Ever-Breastfed**										
Unmatched	0.63	0.74	−0.11	0.03	−4.32 *	0.69	0.76	−0.07	0.04	−1.94 *
Matched	0.66	0.74	−0.08	0.03	−2.51 *	0.69	0.73	−0.04	0.05	−0.83
**Breastfed** **at 6 months**										
Unmatched	0.36	0.52	−0.16	0.03	−4.97 *	0.32	0.48	−0.17	0.05	-3.13 *
Matched	0.40	0.51	−0.11	0.04	−2.52 *	0.32	0.45	−0.13	0.08	−1.67 *

Note: Bootstrapping standard error is 0.05 (with 100 replications). * *p* < 0.05 (one-tailed test)
